# Association between IL-6 polymorphisms and Atopic Dermatitis in Chinese Han children

**DOI:** 10.3389/fped.2023.1156659

**Published:** 2023-03-21

**Authors:** Shuangshuang Huang, Hao Wang, Huiwen Zheng, Wei Li, Jianrong Shi, Chen Shen, Ran Tao

**Affiliations:** ^1^ Department of Clinical Laboratory, Children's Hospital, Zhejiang University School of Medicine, National Clinical Research Center for Child Health, Hangzhou, China; ^2^ Department of Dermatology, Children's Hospital, Zhejiang University School of Medicine, National Clinical Research Center for Child Health, Hangzhou, China; ^3^ Department of Data and Information, Children's Hospital, Zhejiang University School of Medicine, National Clinical Research Center for Child Health, Hangzhou, China; ^4^ Sino-Finland Joint AI Laboratory for Child Health of Zhejiang Province, Hangzhou, China

**Keywords:** IL-6, polymorphisms, atopic dermatitis, susceptibility, inflammatory

## Abstract

**Introduction:**

Atopic Dermatitis (AD) is a chronic inflammatory skin disease that affects almost 20% of children and 2 -10% of adults worldwide. Previous studies revealed that Interleukin-6 (IL-6) plays an essential role in autoimmune and chronic inflammatory diseases. This study aims to investigate the associations between IL-6 polymorphisms and AD.

**Methods:**

Blood samples were collected from 132 AD patients and 100 controls, and single nucleotide polymorphisms (SNPs) in IL-6 (rs2069840 (C/G), rs2066992 (G/T), rs2069837 (A/G) and rs1800796 (G/C)) were analyzed using Multiplex PCR-Based Next Generation Sequencing (NGS).

**Results:**

Results showed that the A/G genotype of IL-6/rs2069837 was significantly associated with a 1.933-fold increased risk of AD compared to those patients with A/A genotype (OR 1.933; 95%CI 1.086-3.438; *p*=0.024). The combined A/G-G/G genotype raised AD risk by 1.856 times compared to patients with the A/A genotype in dominant model (OR: 1.856; 95% CI: 1.056-3.261; *p*=0.030). No association was observed for 3 other SNPs and 4 haplotypes.

**Discussion:**

These findings suggested that the A/G genotype of IL-6/rs2069837 was more susceptible to AD than A/A genotype in Chinese Han children, indicating the risk role of IL-6/rs2069837 in the occurrence of AD.

## 1. Introduction

Atopic Dermatitis (AD), also known as eczema, is a chronic inflammatory skin disease characterized by dry skin, intense itch and relapsing eczema lesions (1), affecting almost 20% of children and 2%–10% of adults worldwide (2). Due to the physical and psychological effects on patients, AD can have a severely impact on the life quality and finances of patients and their families (3, 4). Although the exact pathophysiological mechanism of AD is still unknown, genetic predisposition, epidermal dysfunction, immunological disturbances, and microbial dysbiosis are thought to be the key factors in the onset and progression of AD (2, 5, 6).

Interleukin-6 (IL-6) acts as a pro-inflammatory and anti-inflammatory cytokine that plays a crucial role in the host defense mechanism. Its protein structure consists of four long α-helices, containing two gp130 binding sites and one IL-6Rα binding site (7). The IL-6 family is composed of IL-6, IL-11, IL-27, Oncostatin M (OSM), Ciliary Neurotrophic Factor (CNTF), Cardiotrophin 1 (CT-1), Leukemia Inhibitory Factor (LIF) and Cardiotrophin-Like Cytokine (CLC) (8). These cytokines are grouped into a family due to their common use of the gp130 receptor subunit. However, the IL-6 family cytokines continue to expand and the classification criterion of gp130-containing needs to be revised (9).

Substantial studies have demonstrated that IL-6 is involved in a variety of autoimmune and chronic inflammatory diseases, such as inflammatory bowel disease (IBD), diabetes, rheumatoid arthritis (RA), multiple sclerosis, asthma and most recently, COVID-19 pneumonia (7, 10–13). Furthermore, research has indicated that a functional IL-6 Receptor (IL-6R) variant is a risk factor for persistent AD (14). To date, the association between IL-6 SNPs and AD susceptibility has been studied in Iran, Germany and Czech Republic but not in Chinese Hans (15–17). Therefore, this study aimed to investigate the association between IL-6 SNPs and AD risk in Chinese children.

Maria Carmen Cénit et al. have demonstrated that rs2069840 may influence the development and progression of systemic sclerosis (18). In addition, a Moroccan population study found that rs2069840 was significantly associated with the risk of lung cancer (19). Promoter SNP rs1800796 and intronic SNP rs2066992 were linked to protection against HBV infection and severe COVID-19 (20, 21). Moreover, rs2069837 has been correlated with an increased risk for RA and the G variant was associated with protection against chronic periodontitis in Brazil (22, 23). Given the hypothesis that different inflammatory disorders have common genetic determinants, this study selected four SNPs of rs2069840 (C/G), rs2066992 (G/T), rs2069837 (A/G) and rs1800796 (G/C) to investigate the population-specific susceptibility of IL-6 genetic variation on AD.

## 2. Materials and methods

### 2.1. Subjects

This study surveyed 132 children with AD who visited the dermatology clinic of the Children's Hospital of Zhejiang University School of Medicine from February 2021 to August 2021. Diagnosis of AD was established by dermatologists based on the criteria proposed by Hanifin and Rajka and AD severity was assessed using the Severity Scoring of Atopic Dermatitis (SCORAD) index as mild, moderate or severe (1).

Meanwhile, another 100 healthy controls were recruited from children undergoing routine medical examinations in the hospital health checkup centers. All had no history of atopic or other allergic diseases, and all 232 subjects were of Chinese Han ethnicity. Their demographics and clinical characteristics were presented in Table 1.

**Table 1:** Demographics and clinical characteristics of AD patients and controls.

		Gender			Age		Family history	
Characteristics	Number	Male *n* (%)	Female *n* (%)	*p*-value	Mean ± SD	*p*–value	Patient *n* (%)	*p-*value
Controls	100	66 (66.0)	34 (34.0)	0.131	4.9 ± 1.9	0.232	–	0.636
AD patients	132	73 (55.3)	59 (44.7)		2.6 ± 2.1		100 (75.8)	
* Mild*	47	27 (57.4)	20 (42.6)	0.939	2.6 ± 2.1	0.729	35 (74.5)	
* Moderate*	52	27 (51.9)	25 (48.1)		2.7 ± 2.2		38 (73.1)	
* Severe*	33	19 (59.4)	14 (40.6)		2.5 ± 1.9		27 (84.4)	

AD, atopic dermatitis; SD, standard deviation.

### 2.2. DNA extraction and SNP genotyping

Peripheral blood sample (1 ml) was collected in Ethylene Diamine Tetraacetic Acid (EDTA) anticoagulant tube from all subjects. 200 µl whole blood sample was taken from each subject for human genome DNA extraction using Biospin genomic DNA Extraction Kit (BIOER technology, #BSC06S1). Genomic DNA concentration and purity were detected by Nanodrop 1,000c spectrophotometer (Thermo Scientific, United States). Then extracted DNA samples were stored at −20°C for subsequent experiments.

Four SNPs in IL-6 gene were detected by using Multiplex PCR-Based Next Generation Sequencing technology. The first round of the multiplex PCR was carried out in a Gene Amp PCR System 9,600 (Norwalk, United States) with the following requirements: PCR amplification system was 10 µl, including PCR Buffer (10×) 1 µl, primer (50 nM) 2 µl, dNTP (2.5 mM) 0.8 µl, DNA polymerase (5 U/μl) 0.1 μl, Mg^2+^ (100 mM) 1 μl, genomic DNA 2 μl, ddH_2_O 3.2 μl. 95°C 15 min; 4 cycles of 94°C 30 s, 60°C 10 min and 72°C 30 s; 20 cycles of 94°C 30 s, 60°C 1 min and 72°C 30 s. 1 μl of the first cycle product was used as sample genomic DNA for the second cycle PCR in this state: PCR amplification system was 20 μl, including PCR Buffer (10×) 2 μl, primer (2 μM) 3.6 μl, dNTP (2.5 mM) 0.8 μl, DNA polymerase (5 U/μl) 0.1 μl, Mg^2+^ (100 mM) 1 μl, genomic DNA 9 μl, ddH_2_O 3.6 μl. 95°C 15 min; 5 cycles of 94°C 30 s, 60°C 4 min and 72°C 30 s; 10 cycles of 94°C 30 s, 65°C 1 min and 72°C 30 s. Table 2 lists the primers used for the multiplex PCR. The prepared library was sequenced on the HiSeq X Ten platform (Illumina, United States), and the bwa and samtools pipeline was used for raw data mapping and SNP genotyping.

**Table 2:** Primers for multi-PCR.

SNP ID	Forward primer (5'-3')	Reverse primer (5'-3')
***IL-6***		
rs2069840	TGTATACATATAGATCCAGGCAGC	CCCAGGATGAACTAATTAAACCTG
rs2066992	GACTCAGGATTAAGTAACACACCTAAAG	GTTACATGTCTGGGAAAGAATACC
rs2069837	CTCTGGACTCCATCAGTAAAATTG	TCAGTTTCCTTATCTCCAAAAACC
* *rs1800796	CTTGAAGTAACTGCACGAAATTTG	TTAACAGGCTAGAATTTAGCGTTC

SNP, single nucleotide polymorphism.

### 2.3. Statistical analysis

SPSS software package (version: 22.0) was performed for statistical analysis. Agreement with the Hardy–Weinberg Equilibrium (HWE) for each SNP was tested using a goodness-of-fit Chi-square (*χ*^2^) test. *χ*^2^ or Fisher exact tests were used to compare genotype and allele frequencies. The Odds Ratio (OR) and 95% Confidence Interval (CI) were calculated to estimate the relative risk. Haploview 4.2 software was implemented for haplotype analysis, D' and *r*^2^ calculation, and *p < *0.05 was considered statistically significant.

## 3. Result

### 3.1. Patients' characteristics

The demographic and clinical characteristics of 132 AD patients and 100 healthy controls included in this study were shown in Table 1. The control group consisted of 66 males and 34 females with a mean age of 4.9 ± 1.9 years old, and the case group had 73 males and 59 females with a mean age of 2.6 ± 2.1 years old. No significant difference in mean age and gender was found between cases and control children. Among the cases, 47 (35.6%) were diagnosed as mild, 52 (39.4%) were moderate and 33 (25.0%) were severe in severity. There were no significant differences in gender and mean age among the mild, moderate and severe groups. In addition, 75.8% (100/132) of the children with AD had a family history of allergies, which was not associated with the severity of AD.

### 3.2. Distribution of IL-6 genotypes and alleles in AD patients and controls

The distributions of four IL-6 SNPs were in Hardy–Weinberg equilibrium (HWE) (*p *> 0.05) (data not shown), indicating good population representativeness. The call rate of the four SNPs was 97.84%–99.57%. The genotypes and alleles of each locus were compared between AD patients and controls (Table 3). There were no significant differences in IL-6 genotype and allele frequencies between AD patients and controls (*p *> 0.05).

**Table 3:** Genotype and allele frequencies of *IL-6* SNPs in AD patients and controls.

SNP ID	Genotype	Control	AD	OR (95% CI)	*p*-value
		*n* = 100 (%)	*n* = 132 (%)		
***IL-6***					
rs2069840	C/C	86 (86.9)	118 (89.4)	1.00^Ref^	
	C/G	13 (13.1)	14 (10.6)	0.785 (0.351–1.755)	
	G/G	0 (0)	0 (0)	–	0.555
	C allele	185 (93.4)	250 (94.7)	1.00^Ref^	
	G allele	13 (6.6)	14 (5.3)	0.797 (0.366–1.736)	0.568
rs2066992	G/G	3 (3.2)	8 (6.1)	1.00^Ref^	
	G/T	38 (40.0)	60 (45.4)	0.592 (0.148–2.372)	
	T/T	54 (56.8)	64 (48.5)	0.444 (0.112–1.759)	0.353
	G allele	44 (23.2)	76 (28.8)	1.00^Ref^	
	T allele	146 (76.8)	188 (71.2)	0.745 (0.485–1.146)	0.180
rs2069837	A/A	68 (71.6)	76 (57.6)	1.00^Ref^	
	A/G	25 (26.3)	54 (40.9)	1.933 (1.086–3.438)	
	G/G	2 (2.1)	2 (1.5)	0.895 (0.123-6.526)	0.074
	A allele	161 (84.7)	206 (78.0)	1.00^Ref^	
	G allele	29 (15.3)	58 (22.0)	1.563 (0.957–2.554)	0.075
rs1800796	G/G	3 (3.03)	7 (5.3)	1.00^Ref^	
	G/C	40 (40.4)	61 (46.2)	0.654 (0.160–2.677)	
	C/C	56 (56.6)	64 (48.5)	0.490 (0.121–1.985)	0.402
	G allele	46 (23.2)	75 (28.4)	1.00^Ref^	
	C allele	152 (76.8)	189 (71.6)	0.763 (0.499–1.166)	0.211

AD, atopic dermatitis; SNP, single nucleotide polymorphism; OR, odds ratio; CI, confidence intervals; Ref, reference.

Because the OR of the rs2069837 95% CI: (1.086–3.438) was greater than 1, we further analyzed its genetic models consisting of codominant, dominant and recessive models in AD cases and controls (Table 4). In codominant models, the frequency of A/G genotype was significantly higher in AD patients than in controls (OR: 1.933; 95% CI: 1.086–3.438; *p *= 0.024). This is in contrast to the frequency distribution of A/A genotype in AD patients and controls, suggesting that A/G genotype was associated with a 1.933-fold increased risk for AD compared to patients with A/A genotype. Similarly, the combined A/G-G/G genotype raised AD risk by 1.856 times compared to patients with A/A genotype in the dominant model (OR: 1.856; 95% CI: 1.056–3.261; *p *= 0.030).

**Table 4:** Genotype model analysis of *IL-6* rs2069837 in AD cases and controls.

Genotype model	Control *n* = 100	AD *n* = 132	OR (95% CI)	*p*-value
	*n* (%)	*n* (%)		
**Codominant**				
A/A	68 (71.6)	76 (57.6)	1.00^Ref^	
A/G	25 (26.3)	54 (40.9)	**1.933** **(1.086–3.438)**	**0** **.** **024**
G/G	2 (2.1)	2 (1.5)	0.895 (0.1236.526)	0.074
**Dominant**				
A/A	68 (71.6)	76 (57.6)	1.00^Ref^	
A/G-G/G	27 (28.4)	56 (42.4)	**1.856** **(1.056–3.261)**	**0** **.** **030**
**Recessive**				
A/A-A/G	93 (97.9)	130 (98.5)	1.00^Ref^	
G/G	2 (2.1)	2 (1.5)	0.715 (0.099–5.171)	0.740

Significant differences are shown in bold.

AD, atopic dermatitis; OR, odds ratio; CI, confidence intervals; Ref, reference.

### 3.3. Haplotype analysis

Since IL-6 genes are located close to each other on chromosome 7, we performed the linkage disequilibrium and haplotype analysis. Our results revealed that 4 pairs of IL-6 polymorphisms were in strong linkage disequilibrium (Table 5, D' = 1). As shown in Table 6, four different haplotypes were derived from the observed genotypes, but no statistically significant differences in haplotype distribution were founded between AD patients and controls.

**Table 5:** Linkage disequlibrium analysis between *IL-6* SNPs.

SNP ID	SNP ID	D'	*r* ^2^
rs2069840	rs2066992	1	0.171
rs2069840	rs2069837	1	0.014
rs2069840	rs1800796	1	0.177
rs2066992	rs2069837	1	0.660
rs2066992	rs1800796	1	0.933
rs2069837	rs1800796	1	0.615

SNP, single nucleotide polymorphism.

**Table 6:** Haplotype frequencies of *IL-6* in AD patients and control.

Gene					Frequency controls with haplotype present	Frequency AD with haplotype present	OR (95% CI)	*p* value
*IL-6*	rs2069840	rs2066992	rs2069837	rs1800796				
	C	T	A	C	0.768	0.704	1.00^Ref^	
	C	G	G	G	0.147	0.212	0.59 (0.35–1.00)	0.052
	G	G	A	G	0.065	0.053	1.11 (0.49–2.51)	0.810
	C	G	A	G	0.015	0.011	1.20 (0.23–6.17)	0.830

AD, atopic dermatitis; OR, odds ratio; CI, confidence intervals; Ref, reference.

### 3.4. The distribution of IL-6 genotypes and alleles in mild-to-moderate cases and severe cases

To further investigate whether these IL-6 SNPs were correlated with the severity of AD, the genotypes and alleles of each SNP were compared between mild-to-moderate cases and severe cases (Table 7). There were no significant differences in IL-6 genotype and allele frequencies between them (*p *> 0.05).

**Table 7:** Genotype and allele frequencies of *IL-6* SNPs in patients with mild-to-moderate and severe AD.

SNP ID	Genotype	Mild-to-moderate	Severe	OR (95% CI)	*p*-value
		*n* = 99 (%)	*n* = 33 (%)		
***IL-6***					
rs2069840	C/C	88 (88.89)	30 (90.91)	1.00^Ref^	
	C/G	11 (11.11)	3 (9.09)	0.800 (0.209–3.062)	
	G/G	0 (0)	0 (0)	–	0.745
	C allele	187 (94.44)	63 (95.45)	1.00^Ref^	
	G allele	11 (5.56)	3 (4.55)	0.810 (0.219–2.995)	0.752
rs2066992	G/G	6 (6.06)	2 (6.06)	1.00^Ref^	
	G/T	46 (46.46)	14 (42.42)	0.913 (0.165–5.040)	
	T/T	47 (47.47)	17 (51.52)	1.085 (0.199–5.903)	0.917
	G allele	58 (29.29)	11 (16.67)	1.00^Ref^	
	T allele	140 (70.71)	48 (72.73)	1.808 (0.877–3.726)	0.109
rs2069837	A/A	57 (57.58)	19 (57.58)	1.00^Ref^	
	A/G	40 (40.40)	14 (42.42)	1.933 (1.086–3.438)	
	G/G	2 (2.02)	0 (0.00)	0.895 (0.123–6.526)	0.556
	A allele	154 (77.78)	52 (78.79)	1.00^Ref^	
	G allele	44 (22.22)	14 (21.21)	0.942 (0.478–1.857)	0.864
rs1800796	G/G	5 (5.05)	2 (6.06)	1.00^Ref^	
	G/C	48 (48.48)	13 (39.39)	0.677 (0.118–3.899)	
	C/C	46 (46.46)	18 (54.55)	0.978 (0.174–5.507)	0.662
	G allele	58 (29.29)	17 (25.76)	1.00^Ref^	
	C allele	140 (70.71)	49 (74.24)	1.194 (0.635–2.244)	0.582

AD, atopic dermatitis; SNP, single nucleotide polymorphism; OR, odds ratio; CI, confidence intervals; Ref; reference.

## 4. Discussion

AD is a common chronic inflammatory skin disease, and most people with AD have a family history of allergic disease or atopic diathesis such as rhinitis, conjunctivitis or asthma. This is consistent with our study, in which 75.8% (100/132) of children with AD had a family history of allergies. Although it is known that AD has a genetic background, studies to date have failed to identify the genetic defects that contribute to its development (24).

IL-6 exerts its pleiotropic functions through two main signaling pathways. In the classical signal transduction pathway, IL-6 binds to the IL-6R on the cell membrane and then binds to gp130 on the membrane to activate JAK and initiate intracellular signal transduction. In the trans-signal transduction pathway, the extracellular portion of IL-6R can be hydrolyzed by proteases to form soluble IL-6R (sIL-6R). IL-6 binds to sIL-6R to form a complex, which then binds to gp130 on the cell membrane to initiate intracellular signal transduction. They can then both activate downstream signaling pathways, including JAK-STAT, PI3K/AKT and MAPK, to transmit a variety of biological signals to different tissues and cells (25, 26). Because many cells do not express IL-6R, and all cells can express gp130, cells that cannot respond to the classical signaling pathway can respond to the trans-signaling pathway (27, 28). It is by balancing these two signaling pathways that IL-6 exerts its pro-inflammatory and anti-inflammatory functions. Once dysfunctional, it can cause inflammatory diseases and even cancer (29).

In this study, we investigated the relationship between IL-6 polymorphisms and AD in Chinese Han children. Our study of IL-6/rs2069837 showed that A/G genotype was more susceptible to AD (1.933-fold increased risk) than A/A genotype, and that the combined A/G-G/G genotype raised AD risk by 1.856 times compared to patients with A/A genotype, which is the first to evaluate the associations of IL-6/rs2069837 and the risk of AD in Chinese children. This implies that the A/G genotype may be an AD susceptible genotype and could be used as a potential screening marker. However, the G/G genotype showed no statistically significant difference in AD, probably because the sample size for the G/G genotype was too small (only 2 cases per group). Therefore, an increased sample size is needed for further validation. Meanwhile, other studies have reported IL-6/rs2069837 is associated with susceptibility to RA, osteonecrosis of the femoral head (ONFH) and hepatocellular carcinoma (HCC) in Chinese Han population (22, 30, 31). Bo Gong et al. identified that the A–G variation in IL-6/rs2069837 reduced the expression of IL-6 in the serum to protect against the critical conditions with COVID-19, especially among the males (32). Perhaps, an in-depth insight into the mechanism will lead to better drug targets for the treatment of AD.

Polymorphisms in the IL-6 promoter include rs1800795, rs1800796 and rs1800797. rs1800796 has been reported to be associated with inflammatory disorders such as RA, osteoarthritis (OA), diabetic nephropathy and HCV etc (33–36). Previous studies on IL-6/SNP and AD susceptibility have been conducted on rs1800795(C/G), and showed that the G allele of rs1800795 is more likely to predispose Czechs and Iranian children to AD, whereas in German populations no such association was found (15–17). In contrast, our study investigated another promoter polymorphism, rs1800796, and showed that it was not related to AD susceptibility in Chinese children. These findings suggest heterogeneity in genetic susceptibility across populations and diseases.

Given the limited effect of individual SNP on AD susceptibility, we performed haplotype analysis for these four SNPs. However, no statistically significant differences were found between AD patients and controls (Table 6). Of note, the *p*-value for the C-T-T-T variant haplotype was 0.052, close to the critical value of 0.05. Whether this haplotype is associated with AD susceptibility could be further investigated with a larger sample size. For haplotype analysis, Tao Chen et al. found that C-T-T variant haplotype, represented by IL-6/rs1800796, rs1524107 and rs2066992, was reduced in patients with severe disease, whereas G-C-G wildtype haplotype increased the risk of severe disease. Mechanistically, C-T-T haplotype lose binding of IL-6 introns to CTCF, resulting in a poorer response to inflammatory stimuli, and thus protecting its carriers from severe COVID-19 infection (20).

Finally, we investigated the correlation between IL-6 SNPs and the severity of AD. Our results showed no significant differences between mild to moderate cases and severe cases (Table 7). Nevertheless, Binqing Fu et al. found that pathogenic T cells and inflammatory monocytes triggered an inflammatory storm of IL-6 overproduction, a lethal killer in patients with severe COVID-19 (37). Tocilizumab, which blocks IL-6 receptors, has been shown to be effective in the treatment of severe COVID-19 (38, 39) and several autoimmune inflammatory diseases (40). Whether Tocilizumab is effective in AD requires further investigation into the mechanism of IL-6 and clinical validation.

## 5. Conclusion

In summary, our findings suggest that A/G genotype of IL-6/rs2069837 is more likely to be associated with AD susceptibility than A/A genotype in Chinese Han children, indicating the risk role of IL-6/rs2069837 in the occurrence of AD.

## Data Availability

The data presented in the study are deposited in the figshare repository, accession number https://doi.org/10.6084/m9.figshare.22225282.v1.
